# The Impact of Switching Intention of Telelearning in COVID-19 Epidemic's Era: The Perspective of Push-Pull-Mooring Theory

**DOI:** 10.3389/fpsyg.2021.639589

**Published:** 2021-07-28

**Authors:** Xin Lin, Shih-Wen Chien, Chung-Wen Hung, Shih-Chih Chen, Athapol Ruangkanjanases

**Affiliations:** ^1^School of Economics and Management, Northeast Electric Power University, Jilin, China; ^2^Department of Intelligent Commerce, National Kaohsiung University of Science and Technology, Kaohsiung, Taiwan; ^3^Department of Accounting Information, Southern Taiwan University of Science and Technology, Tainan, Taiwan; ^4^Department of Information Management, National Kaohsiung University of Science and Technology, Kaohsiung, Taiwan; ^5^Chulalongkorn Business School, Chulalongkorn University, Bangkok, Thailand

**Keywords:** COVID-19, push-pull-mooring theory, information system successful model, user satisfaction, switching costs, trust, telelearning

## Abstract

Under the impact of COVID-19, medical telelearning education is increasingly becoming urgent to resolve the contradiction between the physical isolation of medical students and the need for on-site clinical teaching. In this study, the push-pull-mooring (PPM) theory is integrated into a comprehensive model as a conceptual PPM framework: push factors (information system quality and perceived risk), pull factors [telepresence (TP), trust, etc.], mooring factors (switching costs), and switching intention. The results show that most hypotheses were positive, but perceived risk did not influence user satisfaction significantly, and switching costs did not provide the impact on switching intention. This study provides a comprehensive empirical analysis of key factors influencing the choice of distance education by medical students through the integrated multi-model framework.

## Introduction

The COVID-19 pandemic is characterized by high infectivity, high mortality, and limited vaccination. Therefore, many countries have adopted strict confinement policies that have altered basic social behavior patterns due to the need to maintain social distancing and staying at home (Krishnamurthy, 2020). These strict or partial home quarantine policies have led to rapid changes in higher education. The closure of in-class instruction makes all levels of education uncertain, especially clinical training, thereby presenting both opportunities and challenges.

On the one hand, medical education requires both instructors and students to come in close contact with patients, both for diagnosis and to observe how treatment measures affect the development of the disease. This is an irreplaceable link between treatment and teaching in medical education. On the other hand, in clinical shifts, medical students may be exposed to COVID-19 positive patients, and the infected students may transmit the disease. But isolation measures will severely limit the clinical training and discussion of preclinical medical students. Thus, special remote clinical teaching methods must be developed during the current COVID-19 outbreak, and perhaps in the future, by replacing traditional clinical rotation practice with innovative telelearning methods. Innovations in distance education include digital remote technologies and online lectures based on on-site simulations for telelearning, such as clinical treatment videos, virtual audio-visual platforms, real-time live broadcasts of remote consultation networks, and online chat room group discussions (Sahi et al., 2020).

Medical education in China begins with basic preclinical undergraduate teaching for 2 years, followed by clinical rotations, or internships lasting for 2 years, where medical undergraduates are supervised within the treatment groups. In the final year, medical students take the entrance exams for specialized courses (residency internships or postgraduate internships) according to the guidelines of their University (Rose, 2020). Though medical education programs vary widely, clinical training is an indispensable part of all of them. In some countries, such as the United States, the COVID-19 pandemic has come at a time of educational transformation (Rose, 2020). The sharp increase of infection rate forced medical students to stop clinical training altogether and postpone the final examinations. Similar emergency measures have been taken in several schools in Canada, the United Kingdom, and Australia (Rose, 2020). Many medical students have been affected because the most recent class has not yet graduated.

Telelearning, a combination of digital terminals with internetworking technologies, supplements, or replaces an existing education system (Wang et al., 2019). One main advantage of telelearning is that students can study at their convenience, giving them enough time to absorb the knowledge (Almaiah and Alismaiel, 2019). In addition, telelearning can offer a broader range of subjects, and students can have more opportunities to relate with a larger group of telelearners, beyond their in-person social connections (Aldholay et al., 2018). Furthermore, students will be able to communicate with a broader group of experts, increasing their opportunities for self-improvement (Aldholay et al., 2018; Isaac et al., 2019). The final advantage is the broad range of devices that can be used for telelearning, including mobile network terminals (Wang et al., 2019), as well as off-line and wireless learning (Korucu and Alkan, 2011). Therefore, it can efficiently facilitate academic performance (Tan et al., 2007). Telelearning has been incorporated into government policies and projects, encouraging the integration of this learning mode (Isaac et al., 2019).

The push-pull-mooring (PPM) model, which is often used to investigate switching intention, considers some elements that drive individuals to leave their original living place and move to a new location (Liao et al., 2019). PPM recognizes the push factors that force a group to leave, whereas the pull factors draw people to a destination (Fang and Tang, 2017). The mooring factors are those that facilitate or constrain immigration judgments (i.e., switching costs) (Jung et al., 2017). Overall, the PPM provides researchers with a complete three-dimensional framework for switching willingness (Zhang et al., 2014). This study explores three series of elements that affect the intention of Chinese medical students to switch from traditional clinical medical education to online medical telelearning. The results can guide the telelearning of Chinese medical students during the COVID-19 pandemic and afterward. For this, we integrate the DeLone and McLean (D&M) ISS model, switching costs, and various trust factors into PPM, analyzing the three influencing factors in switching intention. The value of incorporating the three series models with the PPM model is confirmed. This extended PPM framework will help and address elements that stimulate the intention of medical students to switch from the traditional clinical rotation teaching mode to online medical telelearning platform services.

## Background and Literature Review

Telelearning is termed as a new learning technology that sends educational instructions and information over the internet through digital devices (i.e., PC terminal servers, portal computers, iPads, and smartphones) (Isaac et al., 2019).

### Pull-Push-Mooring

The PPM migration framework was first proposed as a human migration concept (Moon, 1995; Lee and Turban, 2001) to define population migration from one terrestrial environment to another (Bansal et al., 2005). In essence, PPM indicates how human movement is affected by the following three factors. Push elements are initiated by negative elements, which act to repel people. Pull factors express positive elements at end point of a journey that draw potential immigrants toward it. Mooring effects are supplemental elements that facilitate or constrain immigration judgments according to personal backgrounds. Our theoretical model is built on the use of PPM to interpret the consumer switch intention of an information system, as presented by Bansal et al. (2005). PPM serves as a theoretical foundation for the following two reasons.

First, the intention of consumers to switch services between dissimilar learning platforms can be seen as an immigration behavior (Lin and Wu, 2021; Lin et al., 2021). Second, former studies have confirmed the effectiveness of PPM to explain and interpret switch intention in various immigration scenarios, that is, blogger activities (Chang et al., 2014), network games (Hou et al., 2011), mobile medical platforms (Hwang et al., 2015), mobile messaging applications (Liao et al., 2019), and mobile services (Calvo-Porral and Levy-Mangin, 2015).

One disadvantage to the application of the PPM framework, however, is that even if PPM is appropriate for immigration study (Hsieh et al., 2012), some key elements in relation to qualities, switching costs, and trust may be overlooked when they are applied for switch intention toward online learning services (Fang and Tang, 2017). Thus, this study supplements PPM by considering practical aspects of online learning services.

### Push Factors

The initial D&M ISS model (ISS) defines the success of information systems (Yakubu and Dasuk, 2018). System quality (SYQ) is termed as quality of information processing system that is expressed by the complete function of an information system. Information quality (IQ) is termed as the system output quality of products, including relevance, user-friendliness, adequacy, exactness, etc. Service quality (SEQ) is defined as the characteristics (i.e., approachability, trustworthiness, simplicity, etc.) of the service system that consumers obtain from the information transmission units and technical supporters. User satisfaction is the degree of value produced by IQ (Lee and Woong-Kyu, 2008).

The D&M ISS model is a popular IS theory to evaluate the success of an IS application in various fields. More than 250 articles demonstrate the successful application of IS model (Tahar et al., 2013). For e-Learning, D&M ISS has been used for many aspects of mobile learning systems. Lin (2007) presented a model to study the elements of successful use of an online learning platform by University students, finding that system, information, and service qualities supported use intention through satisfaction. Cheng (2012) combined technology acceptance model (TAM) with the D&M ISS model to study the effect of different qualities of willingness of students to use an e-learning platform. Lwoga (2011) applied ISS to show that system, information, and service qualities were positive elements of usage intention for an e-learning platform (Cheng, 2012).

Although prior powerful evidence showed that ISS can clarify and anticipate elements affecting the usage intention of an information system, few studies have empirically investigated the usage intention of students by combining ISS with the PPM model. Furthermore, perceived risks, such as privacy issues, platform faults, lost keywords, incompatible operating systems, and software, or poor IQ indicate the possibility that students will lose in the process of mobile learning (Chao, 2019).

### Mooring Factors

Mooring factors, which is known as comprising the “intervention barrier,” are connected with elements that promote human migration. These variables are related to individual circumstances, mental factors, values, standards of living, and social impact, which are complementary elements to the push-pull effect of PPM (Kim et al., 2020). Switching costs of the transition can consume time, energy, money, and psychological attention (Burnham et al., 2003; Ye and Potter, 2011). Therefore, switching costs are sacrifices of users when a user switches from a traditional platform to a newly developed platform (Dess et al., 2007). In the transformation process of medical education, even if there is no economic cost involved, there will be procedural, physical, and emotional costs for the users.

Although substantial pull and push factors may exist, users of a digital service may not switch to another digital service due to mooring factors, such as switching costs. Switching costs act as mooring factors for migration (Bhattacherjee and Park, 2013) and have been identified as critical conversion decisions (Cheng, 2012). Switching costs include the irredeemable time and energy invested in the traditional clinical rotation teaching system (sunk costs), the extra training time and energy expended for registering new accounts and profiles for special remote clinical telelearning technologies (learning costs). We need to evaluate the difficulties related to medical telelearning technologies, including loneliness caused by moving home from medical school, decreased discussions with clinical partners, increased reliance on email, and difficulty drawing boundaries between work and entertainment (evaluation costs). Additionally, though the COVID-19 pandemic has allowed most countries to not consider switching costs, how to control the switching costs will gradually become more important as the epidemic abates (Rose, 2020).

### Pull Factors

Push factors refer to negative factors of the place of origin that drive people away, while pull factors refer to positive factors of a destination that draw individuals to it (Bansal et al., 2005). Population migration from an origin to a destination can be regarded as a transformation behavior, and learners changing learning platforms can be also regarded as a transformation behavior. In population migration, a better quality of life in another place can have a major pull effect. Similarly, if another telelearning platform offers a better learning experience, learners will also consider switching to that better learning platform. Learners may be attracted to change learning platforms in search of functions not offered by the current platform, similar to migrants who move to a new place for better jobs or education (Lee, 1966).

With intelligent technology, such as mobile/handheld devices, medical telelearning can enable medical students to access the required healthcare learning at times and locations convenient to them. It can also increase user trust in the technology. In medical telelearning, because of the risk of personal information loss, trust is a major concern.

Telepresence (TP) means a perception of the user of being present at the virtual location of a provider when the two are actually in different locations (Baek et al., 2019). In the absence of direct experience, having TP be like direct experience can better stimulate emotional and intellectual reactions. TP can help encourage medical students to master important aspects of medical treatment because it ensures that telelearning can feel accurate or real, even though students are not physically present.

Para-social interaction (PSI) refers to the association between audience and performers (Horton and Wohl, 1956), which is an intimate illusion of “real” personal association (Dibble et al., 2015), and it can be fostered by telemedicine education technology. If the virtual relationship seems real enough, medical teaching, and the transmission of medical experience will not even be perceived as merely a substitute for in-person teaching. The results of this study, which was conducted in China, showed that PSI was positively correlated with switch intention (Hwang and Zhang, 2018). Social influence (SI) is the extension of important social relations (i.e., relatives, colleagues, or peers) with confidence in a new mobile platform that should be accepted (Venkatesh et al., 2012; Tam and Oliveira, 2016). SI shows the influence of persons on the telelearning technical adaptation of social relations. When consumers choose a new technology, they often consider the opinions of others and if the attitude of others is positive, users are more likely to adopt it.

## Research Model

Many studies have shown that PPM is a useful framework for empirical tests in different fields, such as the switching willingness related to: tourist hotels (Lehto et al., 2015), wireless networking sites (Zhu et al., 2014), web browsers (Ye and Potter, 2011), and cloud medical services (Hwang et al., 2015). Results showed that PPM positively affected switch intention (Chen and Keng, 2019).

Although these studies provide empirical understanding of immigration between virtual online platform services, there has been little attention to the medical learning situation under COVID-19. This study analyzes the switching intention of medical students between remote clinical teaching (online) and traditional clinical rotation practice teaching platforms (offline). It combines the D&M ISS model with the PPM model to overcome the disadvantages (Almaiah and Alismaiel, 2019).

### Push Factors: ISS Model

System quality is the perceived strength of the user for the ease of operation, intensity of connection and learning, and enjoyable use of the system (Petter and McLean, 2009). Although the advantages of information technology and simulation teaching are clear, telelearning has some drawbacks. First and most importantly, all of these tools are complementary to telelearning, not substitutes (Lin et al., 2019). Second, since the establishment of a virtual telelearning environment or simulation laboratory is expensive and time-consuming, it is less suitable for low-income countries. Third, the virtual online emulator will maintain the principle of no contact with patients and social distance between students, whereas the offline human emulator will overlook the demand of social SYQ distance between students (Murphy, 2020a). These disadvantages will easily telelearning by medical students. But students who are forced to return to the clinical rotation mode will incur the risk of infection, and then becoming new sources of infection themselves. Since SYQ may affect user satisfaction, the following hypothesis is proposed:

H1. System quality significantly influences user satisfaction.

Information quality is the intensity that users believe that the information of online learning is measured by a series of indices, constructed by relevance, organization, accuracy, timeliness, and integrity of the information (Cheng, 2012). The success of learning platform design depends on the success of IQ, which is thus crucial for an educational technology system (Almaiah and Alismaiel, 2019).

Clinical learning is best done bedside with “real” patients. In this situation, medical students not only get first-hand experience with clinical discovery of the patients but also understand the dynamics of interaction, psychology, and consultation of the patients. Furthermore, the professional character of the student is often formed by clinical professionals, whom they regard as career examples, and who can transmit ethical views (Sahi et al., 2020). Telelearning can lead to scattered knowledge, where students cannot connect the skills previously learned, which will to the detriment of their future clinical practice. If this defect cannot be ameliorated, medical teachers and students will need to abandon distance education mode and return to traditional clinical rotation when the pandemic is alleviated. Obviously, the quantity of IQ will directly affect user satisfaction of medical telelearning users. Therefore, the following hypothesis is suggested:

H2. Information quality significantly influences user satisfaction.

Service quality is the degree to which users perceive and expect an overall quality of service (Kim et al., 2004). The SEQ features of this research include knowledge quality of assurance, empathy, quick responsiveness, interactivity, and effort to support the telelearning platform by the universities responsible for maintaining the platform (Isaac et al., 2019). Previous studies revealed that SEQ positively influences user satisfaction and usage intention (Mohammadi, 2015; Lwoga and Lwoga, 2017).

Drawbacks of the SEQ offered by the medical telelearning platform include loneliness as a result of returning home from medical school, decreased communication with colleagues, reliance on e-mail, and endless network interference. These shortcomings of SEQ of telelearning can lead to insufficient learning skills, and will compel a return to traditional clinical rotation. Obviously, this will also affect the satisfaction of medical telelearning users. Thus, the following hypothesis is proposed:

H3. Service quality significantly influences user satisfaction.

Perceived risk (PR) is a perception of a person of unclear or unfavorable outcomes of an action (Glover and Benbasat, 2014), and it is crucial in decision-making (Rahman et al., 2019). As a result of the COVID-19 pandemic, medical students have had to withdraw from clinical rotations and professional examinations. Not only is this a financial burden, but it can also cost them jobs in the health care system (Ahmed et al., 2020). Other important perceived risk factors that prevent medical educators from e-learning include time constraints, low technology levels, inadequate infrastructure, and a lack of institutional strategies (Sahi et al., 2020).

Previous research has shown that perceived risk is inversely associated with willingness to use electronic services (Nicolaou and McKnight, 2006), high risk perception may also result in discontinuation (Yang and Lin, 2015). Hanafizadeh et al. (2014) pointed out that the perceived risk of failing the final graduation examination is vital for telelearning services. Therefore, we expect that perceived risk will reduce the willingness of users in using the existing telelearning service and drive users to switch to traditional clinical rotations. Therefore, the following hypothesis is proposed:

H4. Perceived risk significantly influences user satisfaction.

User satisfaction (US) is the degree to which consumers perceive that a system meets their needs (Sanchez-Franco, 2009). Since US is a key factor affecting the adoption of a new system, it is often applied to evaluate the information system (Montesdioca and Maçada, 2015). In telelearning systems, US affects usage intention (Yakubu and Dasuk, 2018) and actual application (Mohammadi, 2015). Through medical telelearning, medical students will record medical histories in virtual classrooms and observe virtual physical examinations. They will also participate in decision-making, patient/family consultation, and implementation planning. Completing medical telelearning interactions as a complement to e-learning will help build a new hybrid learning model that can promote patient participation. The involvement of medical students in this area can benefit both students and also to patients with COVID-19 care planning (Theoret and Ming, 2020). The following hypothesis is suggested:

H5. User satisfaction significantly influences switch intention.

### Mooring Factors: Switching Costs

Numerous studies show that switching costs are mooring effects that influence immigration and switching intention (Zhang et al., 2014; Chen and Keng, 2019; Singh and Reema, 2020). Switching costs include the possible economic, mental, procedural, or behavioral sacrifices during switching (Chang et al., 2014). Previous studies have shown that switching costs will significantly influence the retention rate of consumers (Xu et al., 2014; Zhou, 2016).

Switching costs not only conclude financial costs but also mental and psychological costs (Chang et al., 2014). They are the obstacles a user faces when switching to a new service mode (Lee and Neale, 2012). Researchers believe that switching costs have multiple dimensions, including learning costs, transaction costs, and sunk costs (Burnham et al., 2003).

To improve medical telelearning education during the COVID-19 pandemic, various assessments are necessary. First, updating the content of medical education may be an advantage of medical distance education, though the results of these changes should be confirmed by subsequent assessments (Rose, 2020). Second, clinical competence also relies on dependable assessment implements to guarantee that the skills of medical students provide effective medical care during residency. Third, clinical skills education brings more innovation, flexibility, and investigation in areas such as question-based learning (Wayne et al., 2020). Since evaluation costs are linked with the time and effort required to make a conversion decision (Burnham et al., 2003). Assessing the effectiveness of medical telelearning requires time, effort, and financial investment. Thus, we test the following hypothesis:

H6a. Evaluation cost significantly influences switching cost.

Learning includes time and energy costs to acquire new skills for the effective use of new products or services (Burnham et al., 2003). Learning investment is usually vendor-specific, in that new investments are required by new suppliers. Since medical remote platform suppliers provide various functions for Smartphone or PC versions, medical student users must spend time to become familiar with the functions of a new mobile telelearning platform in order to replace traditional clinical rotations (Cheng et al., 2019).

For medical colleges and universities, starting these e-learning projects means the financial burden of installing, operating, and maintaining the theme. There is also a need to develop infrastructure and recruit staff. This will be a burden on the resources of schools, and school managers may hesitate to invest in e-learning (Dhir et al., 2017).

H6b. Learning cost significantly influences switching cost.

Sunk cost includes previous investments of time, money, and focus (Rusbult, 1980). People will tend to avoid losing previous investments due to terminated activities (Chang et al., 2014). Switching costs include the financial, mental, physical, and emotional sacrifices that may occur before, during, and after service switching (Kim et al., 2004).

We here consider sunk cost as a mooring variable. That is, once users of medical distance education realize what they have invested in traditional clinical rotations, the resulting sunk costs can become a mooring effect (Sun et al., 2017). After COVID-19 forced colleges and universities to adopt telelearning, the government has required new revenue streams for these programs, so as to reduce the need to constantly increase tuition to make up for the income gap, reduce the operating cost (i.e., buildings and dormitories for traditional clinic rotations), and other sunk costs. Accordingly, two hypotheses can be proposed:

H6c. Sunk costs significantly influence switching cost.

H7. Switching cost significantly influences switch intention.

### Pull Factors: Trust

Pull factors encourage potential immigrants to move to new destinations (Chen and Keng, 2019). Jones et al. (2002) defined alternative attraction as the way in which the positive characteristics of competitive service providers support the switching intention of consumers. Other studies have revealed that the need for important functions of social networks to motivate participation in social network-based learning (Hsu and Lu, 2007). TP influences the degree to which users are immersed in the telelearning environment, which can determine the attitudes of medical telelearning users to the service platform (Steuer, 1992; Ye et al., 2020). A high TP network platform reduces the social distance of users by reducing the intermediary illusion of user perception. When medical telelearning students perceive high TP from a telelearning platform, they will perceive the interaction as more “live” (Ye et al., 2020).

During the COVID-19 pandemic, technically assisted learning is critical to provide continuity in clinical teaching. This includes webcasts, video clips, audio recordings, question-based online chat rooms, learning tutorials, and mannequin telelearning simulators. They require the use of telelearning modules (hypermedia and digital images), patient agents such as virtual patients (telelearning clinical examination, procedures, diagnostic skills, and communication skills) and virtual reality telelearning simulators (showing palpation and aid skills). Therefore, this hypothesis postulates:

H8. Telepresence significantly influences trust.

Para-social interaction is the audience reaction to public individuals when the media is controlled by the platform providers and not affected by joint growth (Hsu, 2020). Innovations in medical telelearning technologies are revolutionizing education, allowing individualization of learning, and enhancing interactions between learners and peers (collaborative learning) while changing the role of lecturers. Telelearning can be integrated into traditional medical teaching (Hsu, 2020). This can support the application of continuity learning theory, so that educators will no longer be primarily publishers of content instead of being learning promoters and ability evaluators (Hsu, 2020). Therefore, this hypothesis postulates:

H9. Para-social interaction significantly influences trust.

Social influence (SI) reveals the influence of other important factors on individual consumer intention (Venkatesh et al., 2003). When a key referrer encourages switching from one mobile platform to another platform, a consumer may defer to them before forming any positive impression of the targeted platform (Xu et al., 2014). Therefore, social influence can promote user switching. Several studies have shown that the effect of SI on the intention to use the technology is also significant (Lin and Chang, 2007; Im et al., 2011). In this study, social impact is considered to be “how important a student is considered to be by others, who think that the student advises adopting a telelearning system” (Xu et al., 2014). Therefore, this hypothesis postulates:

H10. Social influence significantly influences trust.

Trust is an experience feeling and experience of cultural background and psychological formation of a person (Lee and Turban, 2001). Consumers who are more trusting may prefer to choose a trustworthy remote service platform rather than lose a precious opportunity (Lin et al., 2021). Therefore, we believe that improving the fidelity of simulation (the accuracy of replication), especially breaking through the cost limit of medical distance learning, the limit of engineering technology, avoiding danger, ethics, psychological measurement requirements, and time limit, and improving the elements of trust are likely to increase users intention to use medical telelearning platform, thus affecting the sustainability of mobile education platform use, more active and effective response to the outbreak of COVID-19. Consequently, a final hypothesis is proposed.

H11. Trust significantly influences switch intention.

**Figure 1:**
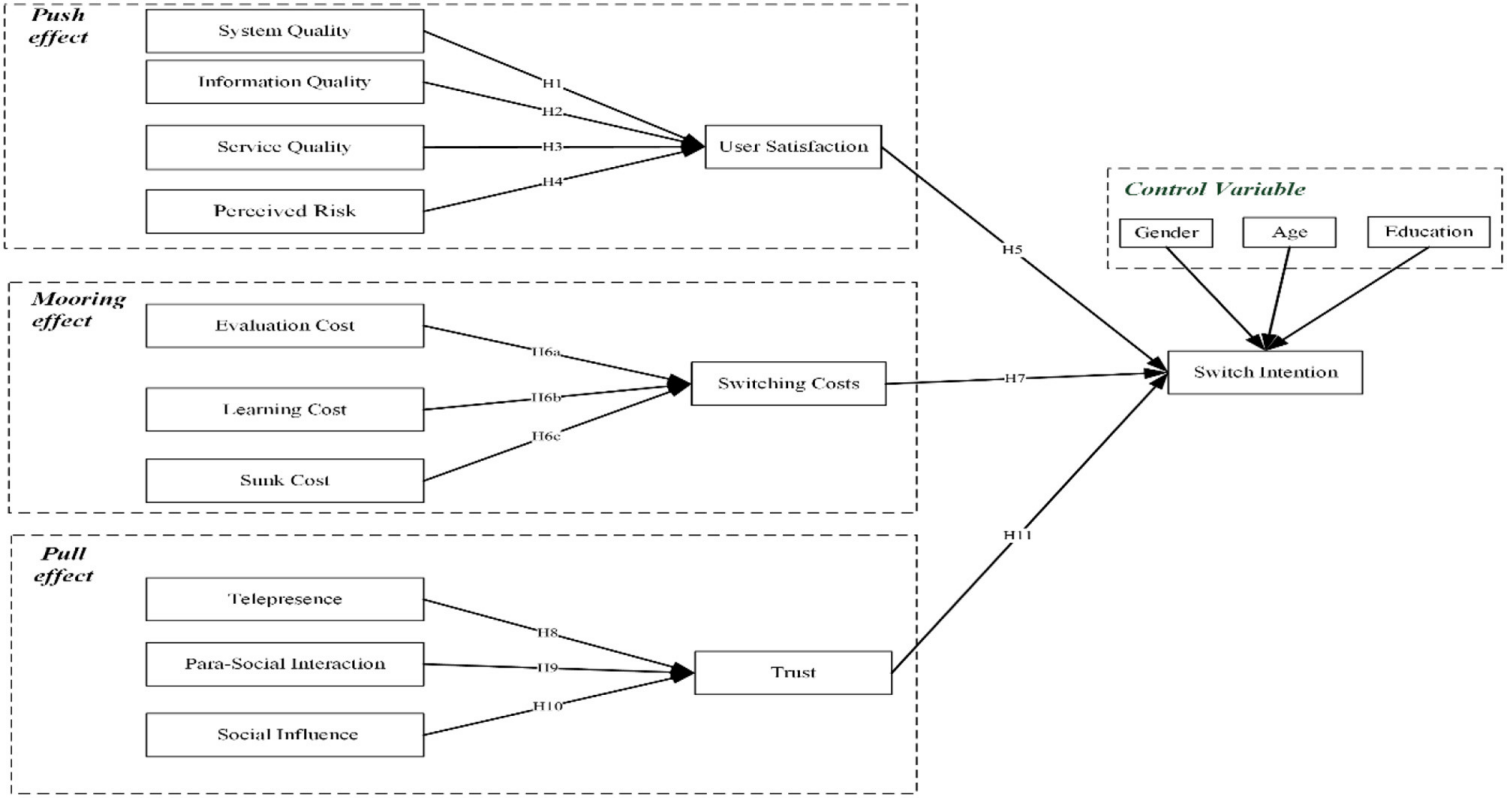
Research model.

According to hypotheses development of this research, we proposed the research model in Figure 1.

## Data Collection and Results

From May 20 to June 5, 2020, we conducted one-on-one interviews with medical students at Jilin University of China for 2 weeks. To ensure the measurement reflected direct behavior practice of the subjects, students who had not participated in telelearning were excluded.

A survey of 390 questionnaires was issued and 322 copies were collected (response rate 82.56%). After discarding 68 responses because of their missing key data or lack of corresponding m-payment experience, a final sample of 310 (96.27%) was used for analysis. The responses were on a five-point Likert scale (Table 1) from “strongly disagree” to “strongly agree.” In the succeeding SEM analysis, IBM SPSS 25.0 was used to check the element structure and inherent relevance of every part. This study uses Cronbach's α to evaluate the structural validity by examining the factor structure and internal correlation of each structure. In order to test the research hypothesis, IBM Amos 24.0 was used to determine the causal relationship by significance value and standard coefficient. Before hypothesis testing, the whole sample is used to analyze the proposed model.

**Table 1:** Sample characteristics.

	**Division**	**Numbers**	**Percentage**
Gender	Male	163	52.58%
	Female	147	47.42%
Age	Below 20	122	39.35%
	20–30	188	60.65%
Education	Undergraduate students	272	87.74%
	Postgraduate students	38	12.26%
Experience	Yes	310	100.0%

### Reliability, Validity, and Measurement Model Evaluation

We evaluated the convergence effect of each measurement item on its related structures, and the standardized load method was used to evaluate the reliability. Composite reliability (CR) was also to measure reliability. In addition, the average variance extracted (AVE) measured the variance of the variable.

As Table 2 shows, CR is higher than 0.80 (Nunnally, 1978), revealing that the optimal validity measures explain the structure of the scale and higher comprehensive consistency level. In addition, convergent validity is measured by three dimensions factors, and the standardized loadings representing the relationship between constructs and indicators is about 0.7 (Gefen et al., 2003); each AVE value was >0.6 (Fornell and Larcker, 1981).

**Table 2:** Convergent validity and reliability.

**Construct**	**Indicators**	**Factor loadings**	**Composite reliability**	**AVE**
SYQ	SYQ 1-4	0.755–0.836	0.866	0.619
IQ	IQ 1-4	0.759–0.813	0.874	0.634
SEQ	SEQ 1-4	0.754–0.878	0.872	0.631
PR	PR 1-4	0.692–0.845	0.853	0.694
EC	EC 1-4	0.785–0.821	0.878	0.644
LC	LC 1-4	0.731–0.789	0.844	0.675
SUC	SUC 1-4	0.760–0.862	0.880	0.648
TP	TP 1-4	0.838–0.912	0.933	0.776
PSI	PSI 1-4	0.795–0.878	0.910	0.717
SI	SI 1-4	0.740–0.848	0.871	0.629
SWC	SWC 1-4	0.673–0.900	0.877	0.644
US	US 1-4	0.805–0.881	0.900	0.694
TR	TR 1-4	0.744–0.824	0.873	0.633
SWI	SWI 1-4	0.818–0.877	0.901	0.703

As shown in Table 3, discriminant validity refers to the difference between the related indicators of the first principle and the second principle (Bagozzi and Phillips, 1991). The test for discriminant validity was performed by evaluating the square root of each variable AVE in each correlation coefficient of construct (Fornell and Larcker, 1981). In addition, for each data, the variance between each structure and the square root of each AVE is greater than any correlation coefficient with respect to the correlation between one structure and another structure, meeting the good discriminant validity of each criterion (Fornell and Larcker, 1981). The correlation between constructs is beyond the diagonal value, which shows that our measurement tool construct validity is satisfactory.

**Table 3:** Discriminant validity.

	**SYQ**	**IQ**	**SEQ**	**PR**	**EC**	**LC**	**SUC**	**TP**	**PSI**	**SI**	**SWC**	**US**	**TR**	**SWI**
SYQ	0.786													
IQ	−0.250	0.795												
SEQ	−0.227	0.167	0.789											
PR	−0.164	−0.019	0.173	0.750										
EC	−0.370	0.350	0.387	0.316	0.818									
LC	−0.348	0.438	0.274	0.214	0.412	0.758								
SUC	0.041	−0.099	−0.082	−0.054	−0.125	−0.157	0.758							
TP	0.166	0.021	0.056	−0.090	−0.118	−0.184	0.346	0.880						
PSI	0.129	0.087	0.139	−0.092	−0.025	−0.105	0.206	0.627	0.872					
SI	0.071	0.092	0.149	−0.007	0.032	−0.029	−0.029	0.228	0.255	0.794				
SWC	−0.059	0.069	0.061	−0.141	−0.011	−0.067	−0.006	0.159	0.130	0.074	0.801			
US	0.173	0.409	0.402	−0.015	0.159	0.136	−0.109	0.319	0.400	0.207	0.168	0.738		
TR	0.089	0.121	0.059	−0.087	−0.076	−0.034	0.043	0.407	0.373	0.302	0.203	0.374	0.793	
SWI	0.165	0.168	0.186	0.027	0.010	−0.054	0.070	0.570	0.599	0.404	0.082	0.530	0.599	0.840

A two-process assessed the selected data. First, the convergence and validity of the algorithm were tested (Anderson and Gerbing, 1988). Then, the integrated framework between the combined models was tested. Finally, the student responses were used to test the proposed structure to test the fit and structure modeling of measurement value. As Table 4 shows, all the model fit indices show that the results of the empirical samples are satisfactory.

**Table 4:** Goodness-of-fit indices for the measurement scales.

**Fit index**	**Recommended value**	**Measurement model**	**Structural model**	**Source**
*Chi-Square*/*d.f*.	<5	1.157	1.199	(Loo and Thorpe, 2000; Schumacker and Lomax, 2004)
CFI	>0.92	0.98	0.97	(Hair et al., 2010)
IFI	>0.90	0.98	0.97	(Bentler and Bonettt, 1980)
TLI	>0.90	0.93	0.93	(Hair et al., 2010)
PGFI	>0.50	0.74	0.76	(Bentler and Bonettt, 1980)
PCFI	>0.50	0.89	0.90	(Bentler and Bonettt, 1980)
PNFI	>0.50	0.79	0.79	(Bentler and Bonettt, 1980)
RMSEA	>0.08	0.08	0.08	(Henry and Stone, 1994)

*CFI, comparative fit index; IFI, incremental fit index; TLI, Tucker-Lewis index; PGFI, parsimony goodness of fit index; PCFI, parsimony comparative fit index; PNFI, parsimony normed fit index; RMSEA, root-mean-squared error of approximation*.

### Hypothesis Verification

**Figure 2:**
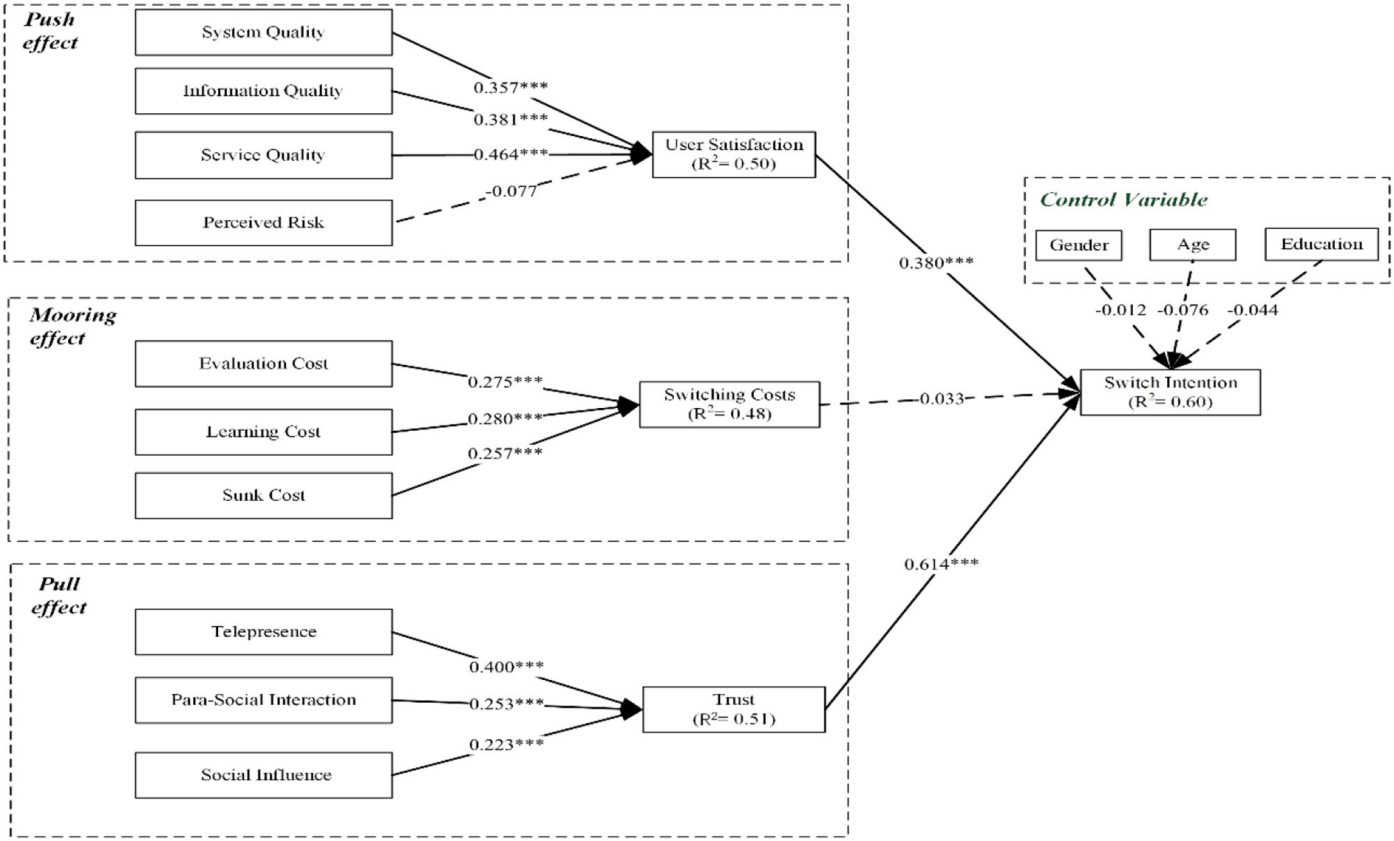
Path analysis of the model. ***p < 0.001.

After testing the measurement suitability and organization of the integrated framework, the path coefficient of the structural model was estimated, as shown in Figure 2, Table 5.

**Table 5:** Results of hypotheses tests.

**Hypothesis**	**Route**	**Estimate**	**S.E**.	***T*-value**	***P***	**Path coefficients**
H1	SYQ → US	0.383	0.062	6.213	^***^	0.357
H2	IQ → US	0.343	0.053	6.438	^***^	0.381
H3	SEQ → US	0.478	0.062	7.677	^***^	0.464
H4	PR → US	−0.079	0.055	−1.429	0.153	−0.077
H5	US → SWI	0.398	0.053	7.511	^***^	0.380
H6a	EC → SWC	0.231	0.059	3.893	^***^	0.275
H6b	LC → SWC	0.224	0.064	3.501	^***^	0.280
H6c	SUC → SWC	0.209	0.061	3.423	^***^	0.257
H7	SWC → SWI	−0.096	0.055	−0.723	0.469	−0.033
H8	TP → TR	0.333	0.056	5.943	^***^	0.400
H9	PSI → TR	0.239	0.065	3.694	^***^	0.253
H10	SI → TR	0.217	0.055	3.910	^***^	0.223
H11	TR → SWI	0.605	0.058	10.499	^***^	0.614

*** *p < 0.001*.

*Control variable 1 (Gender): Beta = −0.012, p > 0.05; Control variable 2 (Age): Beta = −0.076, p > 0.05; Control variable 3 (Education level): Beta = −0.044, p > 0.05*.

The results tested the existence of the comprehensive model as shown in Table 5. Table 5 normalized path coefficients, lists the causal path characteristics, and verifies the combined results of the framework. The basic hypotheses' path coefficients for the proposed comprehensive model were well-evaluated. According to the respective *P*-values, two paths (H4, H7; *p* > 0.05) were not supported, and the other paths were significantly supported. The telelearning switch intention of medical students was influenced by SYQ (β = 0.357), IQ (β = 0.381), SEQ (β = 0.464), PR (β = −0.077), TP (β = 0.400), PSI (β = 0.253), SI (β = 0.223), EC (β = 0.275), LC (β = 0.280),SUC (β = 0.257), US (β = 0.38), TR (β = 0.614), and SWC (β = −0.033) jointly explained 60.0% exploratory power. The influence on medical telelearning platform users showed that user satisfaction, switching costs, and trust account for 50.0, 48.0, and 51.0% of the exploratory variance, respectively, which were related to the 60.0% explanatory rate of integrated structure (Figure 2).

### Mediation Model Verification

Bootstrapping method with bias-corrected confidence interval was used to test the mediating effect (as shown in Table 6). Information system qualities (i.e., SYQ, IQ, and SEQ) indirectly affect switch intention through satisfaction (SYQ->US->SWI: β = 0.14, *p* < 0.001; IQ->US->SWI: β = 0.15, *p* < 0.001; SEQ->US->SWI: β = 0.18, *p* < 0.001). Another mediator, trust, also links the effects of TP, PSI, and social influence to switch intention. However, when the exogenous construct is PR, the mediating effect results is −0.029, and the confidence interval is −0.073 ~ 0.013, including zero. The significance level (*P* = 0.174) indicates that the independent variable PR did not provide the indirect effect on SWI through US. When the independent variable is EC, LC, and SUC; the mediating effect result is −0.009, −0.009, and −0.008; the confidence interval is −0.032~0.013, −0.038~0.014, and −0.030~0.014 including zero. The significance *P* = 0.469, 0.469, and 0.469 indicate that the independent variables EC, LC, and SUC did not affect SWI through SWC.

**Table 6:** Mediating effect analysis.

**Independent variable**	**Mediating variable**	**Dependent variable**	**Mediation effect**	**S.E**.	**LLCI**	**ULCI**	***P*-value**
SYQ	US	SWI	0.135	0.029	0.085	0.199	^***^
IQ	US	SWI	0.145	0.031	0.088	0.206	^***^
SEQ	US	SWI	0.176	0.035	0.111	0.252	^***^
PR	US	SWI	−0.029	0.022	−0.073	0.013	0.153
EC	SWC	SWI	−0.009	0.011	−0.032	0.013	0.469
LC	SWC	SWI	−0.009	0.012	−0.038	0.014	0.469
SUC	SWC	SWI	−0.008	0.011	−0.030	0.014	0.469
TP	TR	SWI	0.246	0.046	0.157	0.332	^***^
PSI	TR	SWI	0.156	0.049	0.060	0.252	^***^
SI	TR	SWI	0.137	0.041	0.056		^***^

*** *p < 0.001*.

## Discussion

### Theoretical Contributions

This study examines which factors can influence, foster, and in turn, enhance the willingness of medical students to continue their telemedicine learning experience. When a telemedicine learning model can provide higher levels of TP, medical students can better focus on their learning activities, providing the most effective telelearning experience (Deci and Ryan, 1985). Similar to Hoffman and Novak (2009), this study combines switching costs and trust with ISS, PPM as a pre-condition of the framework. In particular, this study confirms “telepresence” as an important pull factor in a telelearning environment. Extending the theory of push-pull-mooring, 13 hypotheses for the pre-factors of PPM are proposed in this study.

In terms of theoretical significance, this study contributes to the research on telelearning and acceptance of new information technology innovation for medical students in Chinese universities by studying the acceptance of telelearning in their education under the shadow of the COVID-19 pandemic. The telelearning model proposed in this study includes the key factors in PPM and other factors that have not been studied in previous PPM studies, such as quality factors, transfer costs, and trust, in order to determine the combined influence of these factors on the intention of medical students in Chinese universities to use online learning for clinical training. To our knowledge, previous studies have not studied the impact of these factors in this context. This study also makes a comprehensive evaluation of other factors that affect the use of distance learning. The associated analysis results suggest that the hybrid model is well-suited for use in future research on the use and acceptance of new technologies. In addition to providing useful academic references for future studies in the field of mobile learning, the study also provides theoretical support for the promotion of new telelearning technology.

Based on the updated D&M model with PPM and switching costs, this study helps better understand the effect of quality factors on the behavior patterns of students using a mobile learning system, so as to highlight weak links. The final results present suggestions to improve the distance learning mode of Chinese medical students, suggesting that medical managers should focus on system capabilities, such as SYQ, IQ, and SEQ, as well as TP, PSI, and social influence, which may help to maintain the loyalty of existing telelearning students and attract the trust of potential students.

Several academic implications are presented as follows. First, according to the research results, this study shows that: quality factors (IQ, SYQ, and SEQ) indirectly have a significant impact on the satisfaction and use intention of online learning of college medical students through user satisfaction and use intention. In other words, these three quality factors are the main determinants of use intention and user satisfaction. Therefore, these results show that providing complete in various forms (lectures, courses, assignments, images, and tests) will make telelearning more appealing to students. This study confirms that overall quality has a significant and positive impact on user satisfaction and willingness to use. This means that with a higher quality of distance learning, based on its ease of use, information system features, information reliability, functionality, entire support connected, and flexibility, providing newer, more complete, and relevant information, the Chinese medical University students will appreciate the quality of distance learning.

First, in this study, the weakest mooring effect was the switching cost. The mooring effect (evaluation cost, learning cost, and sunk cost) has a significant negative effect on switching intention. These results maybe because users are familiar with network learning application technologies, such as distance learning systems, distance learning application APPs, and distance learning, and the development of 5G technology can bring better and more stable network applications. Thus, the mooring effect of switching cost has less effect on switching intention than other factors.

Second, according to the research results showed that the impact of perceived risk did not affect user satisfaction, and switching costs did not affect switching intention. Thus, the empirical results revealed that perceived risks and switching costs did not mitigate the impact of user satisfaction and switching intentions. This study focuses on how to design a medical telelearning framework that could foster optimal experiences and further enhanced the willingness of students to continue learning online. The empirical analysis shows that user satisfaction from all kinds of aspects of quality considered was crucial to cultivate the best practice. To attract medical students to participate in telelearning, the platform designer should first create a telelearning environment in which medical users are independently engaged by themselves. This study points out that the platform for telelearning should be built as a transparent layer between the medical telelearners and the telelearning tasks. This can help the medical telelearners to engage with learning activity, and immerse themselves in the telelearning environment. In this sense, the perception of TP is crucial to cultivate the best experience. To enable medical telelearners to participate in their telelearning tasks, the medical telelearning environment should be enhanced so the students feel immersed in the learning experience. For instance, virtual reality (VR), augmented reality (AR), binocular omnidirectional display (BOOM), hybrid reality, and simulation-based education (SBE) are possible choices to for mimicking real medical environments.

Third, prospective theoretical suggestions about telelearning are offered for Chinese medical students. The continued prevalence of COVID-19 has caused unprecedented disruption worldwide and seriously impacted medical education, particularly anatomy, surgery, and clinical training, all of which use in-person training. There is thus greater reliance on telelearning platforms with recorded lectures. If the tools widely utilized in telelearning cannot fully replace some methods and technologies of traditional face-to-face education, the quality of education will be reduced. Consequently, the educational methods and environment urgently need to redesign to effectively incorporate distance learning. The noteworthy solutions are the ones that combine online learning with practical activities provided through VR simulation or advanced artificial intelligence (AI). These virtual or simulated experiences should have a wide range of uses in preparing the next generation of telemedicine, ranging from patient monitoring and healthcare information technology to information analysis collaboration and intelligent diagnostic assistance.

### Managerial Implications

There are several managerial implications of this research. First, it can help the telelearning platform designers recognize the main influencing elements to maintain existing users and attract new learners; Second, these results can help medical academics implement medical telelearning education in colleges and universities through medical telelearning; Third, the results will help governments to accelerate digital distance medical education and improve the efficiency and effectiveness of clinical practice.

Medical students will be in transition for the next several years, and the upcoming exams have already been postponed. For example, the second and third years of medical universities instruction have been suspended and the institutions operating examination centers have temporarily been shut down in America (Murphy, 2020b). Such a delay can discourage students from taking the exams, and if schools are closed for an extended time, their careers may be back. Second, in this global emergency, medical education is challenged. The medical study is a gradual process, in which students must complete tasks targets in a predetermined sequence. If a student missed out on any part of his medical education, it will be more difficult to have a comprehensive understanding. We should therefore adopt new technologies, such as telelearning, to promote continuous development of knowledge and skills in the next generation of medical practitioners (Sahi et al., 2020).

During the COVID-19 pandemic, medical education relies heavily on distance learning to provide continuity in clinical teaching. Telelearning includes webcasts, video clips, audio recordings, question-based learning tutorials online, and digital mannequin simulators. Over the years, these online technologies have been matured. For educational purposes, digital mannequin simulators have been shown to be as effective as real patients (Gillett et al., 2008). The COVID-19 epidemic has clearly had a direct and far-reaching impact on global medical distance education.

Therefore, this study suggests that in this context, special measures are required. In medical telelearning, technological and simulation-based educational innovations (online lectures, video cases, virtual simulators, webcasts, and online chat rooms) should be developed and used. The benefits of medical distance learning to learning terms of safety and epidemic prevention of medical students cannot be overemphasized. Medical telelearning improves the teaching process, which offers more opportunities to take medical courses, decreases overall learning costs, and expands the clinical practice through medical distance learning. The threat of COVID-19 not only brings new challenges to medical education, but also opportunities to test the effectiveness of the traditional education model, and cultivate our ability to embrace rapid change and better integrate new technologies into the medical curriculum. The future of medical education may indeed lie in the successful applications and technologies. Even if these virtual options cannot completely replace practical training, they can support modern medical education in this period of limited physical interaction. However, in order to determine the value and feasibility of this approach, there is a clear need for future research to adopt robust, comprehensive, and culture-specific designs.

## Conclusions, Limitations, and Future Works

Previous studies on migration have shown that the PPM framework is the main pattern in clarifying human switching intention decisions (Bansal et al., 2005). But if only the PPM framework is applied to explain switching intentions of whole subjective and objective factors of medical telelearning, some key contextual factors might be lost. To address deficiencies in theoretical structure and empirical analysis of medical telelearning during the COVID-19 epidemic, three series of factors (i.e., ISS, switching costs, and trust) are combined with PPM to form a complete and integrated model as a conceptual framework. This conceptual framework enhances the lack of explanatory power of the three separation models and further clarifies the subjective and objective factors affecting switching intention. For this, the current study constructed a complete multi-dimensional framework of medical telelearning.

The factors in the extended PPM model of this research concludes: push factors (various qualities of D&M ISS model, perceived risk, and user satisfaction), pull factors (TP, PSI, social influence, and trust), mooring factors (evaluation cost, learning cost, sunk cost, and switching cost), which analyzed the interaction and transformation willingness between different variables (Chen and Keng, 2019). The results showed that 11 of the 13 hypotheses were true, but the impact of perceived risk did not reduce user satisfaction, and switching costs did not hamper switching intention.

Several limitations regarding this study are worth pursuing in the future. First, future research can consider the age, experience, and other factors of medical telelearning teachers into the theoretical model as regulatory factors to investigate whether there are differences between different samples of students and teachers according to these characteristics. Second, this study includes only Chinese medical students. To enhance the generalizability of the findings, we hope to compare research results of different countries. Third, we combine D&M ISS, switching costs, and Trust with PPM to confirm which elements affect willingness to use mobile payments. Fourth, due to the limitations of distance learning system development technology at this stage, it is suggested that future developers use more advanced VR and other remote vision technologies to make up for physical isolation and lack of on-site clinical practice teaching of medical students. Future research can adopt perceived value, ease of use, and a task-technology-fit model to test other factors on stimulating switch intention of medical telelearning education users. Future research can adopt perceived value, ease of use, task-technology-fit model, etc., to test other factors on stimulating switch intention of medical telelearning education users. Future SEM research should test the switch willingness of users in using medical telelearning platforms from a more comprehensive perspective.

## Data Availability Statement

The raw data supporting the conclusions of this article will be made available by the authors, without undue reservation.

## Author Contributions

XL and S-CC: conceptualization. S-WC, S-CC, and AR: methodology. XL: formal analysis and investigation. XL, S-WC, C-WH, S-CC, and AR: writing—original draft preparation. S-CC and AR: writing—review and editing. AR: visualization. All authors have read and agreed to the published version of the manuscript.

## Conflict of Interest

The authors declare that the research was conducted in the absence of any commercial or financial relationships that could be construed as a potential conflict of interest.

## Publisher's Note

All claims expressed in this article are solely those of the authors and do not necessarily represent those of their affiliated organizations, or those of the publisher, the editors and the reviewers. Any product that may be evaluated in this article, or claim that may be made by its manufacturer, is not guaranteed or endorsed by the publisher.

## References

[B1] Ahmed H. Allaf M. Elghazaly H. (2020). COVID-19 and medical education. Lancet Infect. Dis. 15, 223–234. 10.1016/S1473-3099(20)30226-7 PMC7270510 32213335

[B2] Aldholay A. Isaac O. Abdullah Z. Ramayah T. (2018). The role of transformational leadership as a mediating variable in DeLone and McLean information system success model: the context of online learning usage in Yemen. Telemat. Informat. 35, 1421–1437. 10.1016/j.tele.2018.03.012

[B3] Almaiah M. A. Alismaiel O. A. (2019). Examination of factors influencing the use of mobile learning system: an empirical study. Educ. Informat. Technol. 24, 885–909. 10.1007/s10639-018-9810-7

[B4] Anderson J. C. Gerbing D. W. (1988). Structural equation modelling in practice: a review and recommended two-step approach. Psychol. Bull. 103, 411–423. 10.1037/0033-2909.103.3.411

[B5] Baek E. Lee H. K. Choo H. J. (2019). Cross-border online shopping experiences of Chinese shoppers. Asia Pacific J. Market. Logistics 32, 366–385. 10.1108/APJML-03-2018-0117

[B6] Bagozzi R. P. Phillips L. W. (1991). Assessing construct validity in organizational research. Admin. Sci. Quart. 36, 421–430. 10.2307/2393203

[B7] Bansal H. S. Taylor S. F. St. James Y. (2005). “Migrating” to new service providers: toward a unifying framework of consumers' switching behaviors. J. Acad. Market. Sci. 33, 96–115. 10.1177/0092070304267928

[B8] Bentler P. M. Bonettt D. (1980). Significance tests and goodness-of- fit in the analysis of covariance structures. Psychol. Bull. 88, 588–606. 10.1037/0033-2909.88.3.588

[B9] Bhattacherjee A. Park S. C. (2013). Why end-users move to the cloud: a migration-theoretic analysis. Europ. J. Informat. Syst. 23, 357–372. 10.1057/ejis.2013.1

[B10] Burnham T. A. Frels J. K. Mahajan V. (2003). Consumer switching costs: a typology, antecedents, and consequences. J. Acad. Market. Sci. 31, 109–126. 10.1177/0092070302250897

[B11] Calvo-Porral C. Levy-Mangin J.-P. (2015). Switching behavior and customer satisfaction in mobile services: analyzing virtual and traditional operators. Comput. Human Behav. 49, 532–540. 10.1016/j.chb.2015.03.057

[B12] Chang I. Liu C. C. Chen K. (2014). The push, pull and mooring effects in virtual migration for social networking sites. Informat. Syst. J. 24, 323–346. 10.1111/isj.12030

[B13] Chao C. M. (2019). Factors determining the behavioral intention to use mobile learning: an application and extension of the UTAUT model. Front. Psychol. 10:1652. 10.3389/fpsyg.2019.01652 31379679 PMC6646805

[B14] Chen Y.-H. Keng C.-J. (2019). Utilizing the Push-Pull-Mooring-Habit framework to explore users' intention to switch from offline to online real-person English learning platform. Internet Res. 29, 167–193. 10.1108/IntR-09-2017-0343

[B15] Cheng S. Lee S. J. Choi B. (2019). An empirical investigation of users' voluntary switching intention for mobile personal cloud storage services based on the push-pull-mooring framework. Comput. Human Behav. 92, 198–215. 10.1016/j.chb.2018.10.035

[B16] Cheng Y. (2012). Effects of quality antecedents on e-learning acceptance. Internet Res. 22, 361–390. 10.1108/10662241211235699

[B17] Deci E. L. Ryan R. M. (1985). Intrinsic motivation and self-determination in human behavior. Springer. 10.1007/978-1-4899-2271-7

[B18] Dess G. Lumpkin G. Eisner A. (2007). Strategic Management: Text and Cases, 3rd Edn. New York, NY: McGraw-Hill Irwin.

[B19] Dhir S. K. Verma D. Batta M. Mishra D. (2017). E-Learning in Medical Education in India. Indian Pediatr. 54, 871–878. 10.1007/s13312-017-1152-9 29120336

[B20] Dibble J. L. Hartmann T. Rosaen S. F. (2015). Parasocial interaction and parasocial relationship: conceptual clarification and a critical assessment of measures. Hum. Commun. Res. 42, 21–44. 10.1111/hcre.12063

[B21] Fang Y. H. Tang K. (2017). Involuntary migration in cyberspaces: the case of MSN messenger discontinuation. Telemat. Inform. 34, 177–193. 10.1016/j.tele.2016.05.004

[B22] Fornell C. Larcker D. F. (1981). Evaluating structural equation models with unobservable variables and measurement error. J. Market. Res. 18, 39–50. 10.1177/002224378101800104

[B23] Gefen D. Straub D. Boudreau M. (2003). Structural equation modeling and regression: guidelines for research practice. Commun. Assoc. Informat. Syst. 4, 56–79. 10.17705/1CAIS.00407

[B24] Gillett B. Peckler B. Sinert R. Onkst C. Nabors S. Issley S.. (2008). Simulation in a disaster drill: comparison of high–fidelity simulators versus trained actors. Acad. Emerg. Med. 15, 1144–1151. 10.1111/j.1553-2712.2008.00198.x 18717651

[B25] Glover S. Benbasat I. (2014). A comprehensive model of perceived risk of e-commerce transactions. Int. J. Electron. Commerce 15, 47–78. 10.2753/JEC1086-4415150202

[B26] Hair J. F. Black W. C. Babin B. J. Anderson R. E. (2010). Multi-variate data analysis: a global perspective (7th ed.). Upper Saddle River, NJ: Pearson Prentice Hall.

[B27] Hanafizadeh P. Behboudi M. Koshksaray A. A. Tabar M. J. S. (2014). Mobile-banking adoption by Iranian bank clients. Telemat. Inform. 31, 62–78. 10.1016/j.tele.2012.11.001

[B28] Henry J. W. Stone R. W. (1994). A structural equation model of end-user satisfaction with a computer-based medical information system. Inform. Resour. Manage. J. 7, 21–33. 10.4018/irmj.1994070102

[B29] Hoffman D. L. Novak T. P. (2009). Flow online: lessons learned and future prospects. J. Interact. Market. 23, 23–34. 10.1016/j.intmar.2008.10.003

[B30] Horton D. Wohl R. R. (1956). Mass communication and para-social interaction. Psychiatry 19, 215–229. 10.1080/00332747.1956.11023049 13359569

[B31] Hou A. C. Y. Chern C.-C. Chen H.-G. Chen Y.-C. (2011). cation and para-social interaction. Psychiatr MMORPG switching through human migration theory. Comput. Hum. Behav. 27, 1892–1903. 10.1016/j.chb.2011.04.013

[B32] Hsieh J.-K. Hsieh Y.-C. Chiu H.-C. Feng Y.-C. (2012). Post-adoption switching behavior for online service substitutes: a perspective of the push–pull–mooring framework. Comput. Human Behav. 28, 1912–1920. 10.1016/j.chb.2012.05.010

[B33] Hsu C.-L. (2020). How vloggers embrace their viewers: focusing on the roles of para-social interactions and flow experience. Telemat. Informat. 49, 101–123. 10.1016/j.tele.2020.101364

[B34] Hsu C. L. Lu H. P. (2007). Consumer behavior in online game communities: a motivational factor perspective. Comput. Human Behav. 23, 1642–1659. 10.1016/j.chb.2005.09.001

[B35] Hwang G. J. Lai C. L. Wang S. Y. (2015). Seamless flipped learning: a mobile technology-enhanced flipped classroom with effective learning strategies. J. Comput. Educat. 2, 449–473. 10.1007/s40692-015-0043-0

[B36] Hwang K. Zhang Q. (2018). Influence of parasocial relationship between digital celebrities and their followers on followers' purchase and electronic word-of-mouth intentions, and persuasion knowledge. Comput. Human Behav. 87, 155–173. 10.1016/j.chb.2018.05.029

[B37] Im I. Hong S. Kang M. (2011). An international comparison of technological adoption: testing the UTAUT model. Informat. Manage. 48, 1–8. 10.1016/j.im.2010.09.001

[B38] Isaac O. Aldholay A. Abdullah Z. Ramayah T. (2019). Online learning usage within Yemeni higher education: the role of compatibility and task- technology fit as mediating variables in the IS success model. Comput. Educ. 136, 113–129. 10.1016/j.compedu.2019.02.012

[B39] Jones M. A. Mothersbaugh D. L. Beatty S. E. (2002). Why customers stay: measuring the underlying dimensions of services switching costs and managing their differential strategic outcomes. J. Bus. Res. 55, 71–82. 10.1016/S0148-2963(00)00168-5

[B40] Jung J. Han H. Oh M. (2017). Travelers' switching behavior in the airline industry from the perspective of the push-pull-mooring framework. Tour. Manage. 59, 139–153. 10.1016/j.tourman.2016.07.018

[B41] Kim M. K. Park M. C. Jeong D. H. (2004). The effects of customer satisfaction and switching barrier on customer loyalty in Korean mobile telecommunication services. Telecommun. Policy 28, 145–159. 10.1016/j.telpol.2003.12.003

[B42] Kim S. Choi M. J. Choi J. S. (2020). Empirical study on the factors affecting individuals' switching intention to augmented/virtual reality content services based on push-pull-mooring theory. Information 11:25. 10.3390/info11010025

[B43] Korucu A. T. Alkan A. (2011). Differences between m-learning (mobile learning) and e-learning, basic terminology and usage of m-learning in education. Procedia Soc. Behav. Sci. 15, 1925–1930. 10.1016/j.sbspro.2011.04.029

[B44] Krishnamurthy S. (2020). The future of business education: a commentary in the shadow of the Covid-19 pandemic. J. Bus. Res. 117, 1–5. 10.1016/j.jbusres.2020.05.034 32501309 PMC7241349

[B45] Lee E. S. (1966). A theory of migration. Demography 3, 47–57. 10.2307/2060063

[B46] Lee J.-K. Woong-Kyu L. (2008). The relationship of e-Learner's self-regulatory efficacy and perception of e- Learning environmental quality. Comput. Human Behav. 24, 32–47. 10.1016/j.chb.2006.12.001

[B47] Lee M. K. O. Turban E. (2001). A trust model for consumer internet shopping. Int. J. Electronic Commerce 6, 75–91. 10.1080/10864415.2001.11044227

[B48] Lee R. N. Neale L. (2012). Interactions and consequences of inertia and switching costs. J. Serv. Market. 26, 365–374. 10.1108/08876041211245281

[B49] Lehto X. Y. Oun-Joung P. Gordon S. E. (2015). Migrating to new hotels: a comparison of antecedents of business and leisure travelers' hotel switching intentions. J. Qual. Assurance Hos. Tourism 16, 235–258. 10.1080/1528008X.2014.925787

[B50] Liao Y. W. Huang Y. M. Huang S. H. Chen H. C. Wei C. W. (2019). Exploring the switching intention of learners on social network-based learning platforms: a perspective of the push–pull–mooring model. EURASIA J. Mathemat. Sci. Technol. Educ. 15:em1747. 10.29333/ejmste/108483

[B51] Lin H. F. (2007). Measuring online learning systems success: applying the updated DeLone and McLean model. Cyberpsychol. Behav. 10, 817–820. 10.1089/cpb.2007.9948 18085970

[B52] Lin I. C. Chang C. C. (2007). A practical electronic payment system for message delivery service in the mobile environment. Wireless Personal Commun. 42, 247–261. 10.1007/s11277-006-9176-9

[B53] Lin X. Chang S. C. Chou T. H. Chen S. C. Ruangkanjanases A. (2021). Consumers' intention to adopt blockchain food traceability technology towards organic food products. Int. J. Environ. Res. Public Health 18:135. 10.3390/ijerph18030912 33494321 PMC7908134

[B54] Lin X. Wu R. Z. (2021). An empirical study on the dairy product consumers' intention to adopt the food traceability's technology: push-pull-mooring model integrated by D&M ISS Model and TPB with ITM. Front. Psychol. 11:612889. 10.3389/fpsyg.2020.612889 33519633 PMC7843444

[B55] Lin X. Wu R. Z. Lim Y. T. Han J. P. Chen S. C. (2019). Understanding the sustainable usage intention of mobile payment technology in korea: cross-countries comparison of Chinese and Korean users. Sustainability 11, 23–46. 10.3390/su11195532

[B56] Loo R. Thorpe K. (2000). Confirmatory factor analyses of the full and short versions of the Marlowe-Crowne Social Desirability Scale. J. Soc. Psychol. 140, 628–635. 10.1080/00224540009600503 11059209

[B57] Lwoga E. T. (2011). Making web 2.0 technologies work for higher learning institutions in Africa. Campus-Wide Informat. Syst. 29, 90–107. 10.1108/10650741211212359

[B58] Lwoga E. T. Lwoga N. B. (2017). User acceptance of mobile payment: the effects of user-centric security, system characteristics and gender. Electron. J. Informati. Syst. Deve. Countries 81, 145–162. 10.1002/j.1681-4835.2017.tb00595.x

[B59] Mohammadi H. (2015). Social and individual antecedents of m-learning adoption in Iran. Comput. Human Behav. 49, 191–207. 10.1016/j.chb.2015.03.006

[B60] Montesdioca G. P. Z. Maçada A. C. G. (2015). Measuring user satisfaction with information security practices. Comput. Secur. 48, 267–280. 10.1016/j.cose.2014.10.015

[B61] Moon B. (1995). Paradigms in migration research: exploring ‘moorings'as a schema. Prog. Hum. Geogr. 19, 504–524. 10.1177/030913259501900404 12347395

[B62] Murphy B. (2020a). Four questions medical students are asking on the COVID-19 pandemic. Public Health 3, 1–14. 34031272

[B63] Murphy B. (2020b). Online learning during COVID-19: Tips to help med students succeed. Am. Med. Assoc. 1–5.

[B64] Nicolaou A. I. McKnight D. H. (2006). Perceived information quality in data exchanges: effects on risk, trust, and intention to use. Informat. Syst. Res. 17, 332–351. 10.1287/isre.1060.0103

[B65] Nunnally J. C. (1978). Psychometric Theory. New York, NY: McGraw-Hill.

[B66] Petter S. McLean E. R. (2009). A meta-analytic assessment of the DeLone and McLean IS success model: an examination of IS success at the individual level. Informat. Manage. 46, 159–166. 10.1016/j.im.2008.12.006

[B67] Rahman S. Thiagarajan R. Louis N. (2019). Impact of social media use on student satisfaction in Higher Education. Higher Educ. Quarte. 11, 71–89. 10.1111/hequ.12228

[B68] Rose S. (2020). Medical student education in the time of COVID-19. JAMA 12, 83–84. 10.1001/jama.2020.5227 32232420

[B69] Rusbult C. E. (1980). Commitment and satisfaction in romantic associations: a test of the investment model. J. Exp. Soc. Psychol. 16, 172–186. 10.1016/0022-1031(80)90007-4

[B70] Sahi P. K. Mishra D. Singh T. (2020). Medical education amid the COVID-19 pandemic. Indian Pediatr. 57, 652–657. 10.1007/s13312-020-1894-7 PMC7387262 32412913

[B71] Sanchez-Franco M. J. (2009). The moderating effects of involvement on the relationships between satisfaction, trust and commitment in e-Banking. J. Interact. Market. 23, 247–258. 10.1016/j.intmar.2009.04.007

[B72] Schumacker R. E. Lomax R. G. (2004). A Beginner's Guide to Structural Equation Modeling. New York, NY: Psychology Press. 10.4324/9781410610904

[B73] Singh R. Reema S. (2020). Why do online grocery shoppers switch? An empirical investigation of drivers of switching in online grocery. J. Retail. Consumer Serv. 53, 188–199. 10.1016/j.jretconser.2019.101962

[B74] Steuer J. (1992). Defining virtual reality: dimensions determining telepresence. J. Commun. 42, 73–93. 10.1111/j.1460-2466.1992.tb00812.x

[B75] Sun Y. Liu D. Chen S. Wu X. Shen X. L. Zhang X. (2017). Understanding users' switching behavior of mobile instant messaging applications: an empirical study from the perspective of push-pull-mooring framework. Comput. Human Behav. 75, 727–738. 10.1016/j.chb.2017.06.014

[B76] Tahar N. F. Mokhtar R. Jaafar N. H. Zamani N. D. Sukiman S. A. Ismail Z. (2013). Students' satisfaction on blended learning: the use of factor analysis, in IEEE Conference on e-Learning, e-Management, and e-Services (IC3e) (Kuching: IEEE), 51–56. 10.1109/IC3e.2013.6735965

[B77] Tam C. Oliveira T. (2016). Understanding the impact of m-banking on individual performance: DeLone & McLean and TTF perspective. Comput. Human Behav. 61, 233–244. 10.1016/j.chb.2016.03.016

[B78] Tan T.-H. Liu T.-Y. Chang C.-C. (2007). Development and evaluation of an RFID-based ubiquitous learning environment for outdoor learning. Interact. Learn. Environ. 15, 253–269. 10.1080/10494820701281431

[B79] Theoret C. Ming X. (2020). Our education, our concerns: the impact on medical student education of COVID-19. Med. Educ. 54, 591–592. 10.1111/medu.14181 32310318 PMC7264564

[B80] Venkatesh V. Morris M. G. Davis G. B. Davis F. D. (2003). User acceptance of information technology: toward a unified view. MIS Quart. 27, 425–478. 10.2307/30036540

[B81] Venkatesh V. T. James Y. L. Xin X. (2012). Consumer Acceptance and Use of Information Technology: Extending the Unified Theory of Acceptance and Use of Technology. MIS Quart. 36, 157–178. 10.2307/41410412

[B82] Wang Y. Y. Wang Y. S. Lin H. H. Tsai T. H. (2019). Developing and validating a model for assessing paid mobile learning app success. Interact. Learn. Environ. 27, 458–477. 10.1080/10494820.2018.1484773

[B83] Wayne D. B. Green M. Neilson E. G. (2020). Medical education in the time of COVID-19. Sci. Adv. 9, 80–96. 10.1126/sciadv.abc7110 PMC7399480 32789183

[B84] Xu Y. Yang Y. Cheng Z. Lim J. (2014). Retaining and attracting users in social networking services: an empirical investigation of cyber migration. J. Strategic Informat. Syst. 23, 239–253. 10.1016/j.jsis.2014.03.002

[B85] Yakubu N. Dasuk S. I. (2018). Assessing eLearning systems success in Nigeria: an application of the DeLone and McLean information systems success model. J. Inform. Technol. Educ. 17, 183–203. 10.28945/4077

[B86] Yang H.-L. Lin S.-L. (2015). User continuance intention to use cloud storage service. Comput. Human Behav. 52, 219–232. 10.1016/j.chb.2015.05.057

[B87] Ye C. Potter R. (2011). The role of habit in post-adoption switching of personal information technologies: an empirical investigation. Commun. Assoc. Informati. Syst. 28, 585–610. 10.17705/1CAIS.02835

[B88] Ye S. L. Sut I. Shen H. L. Xiao H. G. (2020). Social presence, telepresence, and customers' intention to purchase online peer-to-peer accommodation. J. Hosp. Tourism Manage. 42, 119–129. 10.1016/j.jhtm.2019.11.008

[B89] Zhang K. Christy M. K. Lee M. K. O. (2014). Online service switching behavior: the case of blog service providers. J. Electron. Commerce Res. 13, 184–197. 10.1016/j.elerap.2013.12.001

[B90] Zhou T. (2016). Examining user switch between mobile stores: a push-pull-mooring perspective. Informat. Resour. Manage. J. 29, 1–13. 10.4018/978-1-4666-9845-1.ch056

[B91] Zhu D. H. Chang Y. P. Luo J. J. Li X. (2014). Understanding the adoption of location-based recommendation agents among active users of social networking sites. Inf. Process. Manag. 50, 675–682. 10.1016/j.ipm.2014.04.010

